# Concordance of laboratory assays for claudin 18.2 in gastric cancer tissue samples: independent proficiency testing and a descriptive non-interventional study

**DOI:** 10.1007/s00428-025-04138-x

**Published:** 2025-06-28

**Authors:** Christoph Röcken, Anne Kathrin Höhn, Jens Neumann, Hans-Ulrich Schildhaus, Stephan Singer, Till S. Clauditz, Alexander Quaas, Korinna Jöhrens

**Affiliations:** 1 Department of Pathology, University Hospital Schleswig-Holstein, Campus Kiel, Arnold-Heller-Str 3, 24105 Kiel, Germany; 2 Universität Leipzig, Haus G, Liebigstraße 26, 04103 Leipzig, Germany; 3 Institute of Pathology, Medical Faculty, Ludwig Maximilian University Munich, Thalkirchnerstr. 36, 80337 Munich, Germany; 4 Discovery Life Sciences & Institute of Pathology Nordhessen, Germaniastraße 7, 34119 Kassel, Germany; 5 Institute of Pathology, University Hospital Tübingen, Liebermeisterstr. 8, 72076 Tübingen, Germany; 6 Center for Diagnostics, Institute of Pathology, University Medical Center Hamburg-Eppendorf, Martinistraße 52, 20246 Hamburg, Germany; 7 Institute of Pathology, Cologne University Hospital, Kerpener Str. 62, 50937 Köln, Germany; 8 Qualitätssicherungs-Initiative Pathologie QuIP GmbH, Reinhardtstraße 1, 10117 Berlin, Germany

**Keywords:** Antibody, Assay, Biomarker, Claudin 18.2, Gastric/gastroesophageal junction cancer, Proficiency test

## Abstract

Biomarker testing is vital for personalized cancer treatment with improved efficacy based on genotype and biomarker expression. This prospective, in vitro, non-interventional study accompanied an independent proficiency test to descriptively analyze the results of immunohistochemical claudin 18.2 (CLDN18.2) testing in gastric cancer tissue samples by laboratories in Germany, Austria, and Switzerland, using assays independently established in their institutions. CLDN18.2 is a biomarker and drug target for locally advanced unresectable/metastatic gastric and pancreatic cancer. During an open proficiency test (OPT), laboratories were provided with 10 gastric cancer tissue samples (cases) for staining, interpretation, and scoring by a pathologist. Results were submitted with stained tumor cell percentage and a binary CLDN18.2-positive/negative result for each sample (cut-off: ≥ 75% of tumor cells expressing membranous CLDN18 with ≥ 2+ [moderate to strong] staining intensity). Successful participation was defined as ≥ 90% of cases evaluated correctly. Overall proficiency of the OPT was acceptable if ≥ 80% of laboratories participated successfully. Fifty-four institutes registered for the OPT, and one resigned. Forty-two of 53 (79.2%) institutes participated successfully. The main source of errors was false negative results (false negative rate, 12%; false positive rate, 1%). Overall accuracy was 91%. The CLDN18 (43-14A) clone was used most frequently, with optimal staining results and minor interpretation problems observed. Two antibodies were associated with false negative results (ZR451 [Zeta/Zytomed]; EPR19202 [Abcam]). Staining quality was the major factor for successful analysis; interpretation errors were not significant. Ongoing quality assurance will enable establishment of appropriate staining protocols and accurate CLDN18.2 interpretation, thereby facilitating individually optimized treatment.

**Supplementary Information:**

The online version contains supplementary material available at 10.1007/s00428-025-04138-x.

## Introduction

Gastric cancer was the fifth leading cause of cancer-related deaths worldwide in 2022, with an incidence of nearly 1 million cases annually [1]. Claudin 18.2 (CLDN18.2), an isoform of claudin 18 (CLDN18), is a biomarker and drug target for gastric cancer [2, 3]. CLDN18 is a highly specific tight junction protein located in the outer cell membrane of gastric mucosa cells [2–4]. CLDN18.2 is normally expressed in the stomach and frequently expressed in gastric/gastroesophageal junction (G/GEJ) adenocarcinoma and other cancers [2, 4, 5]. In the normal gastric mucosa, CLDN18.2 is not easily accessible due to its location in the tight junction, but with malignant transformation, the junction is disrupted and the target epitope may become more exposed, increasing its accessibility [6].

Zolbetuximab, an antibody that targets CLDN18.2, is a novel treatment for CLDN18.2-positive, human epidermal growth factor receptor 2 (HER2)-negative, locally advanced unresectable or metastatic G/GEJ cancer [7, 8]. Zolbetuximab is approved in the United States, Europe, United Kingdom, South Korea, China, Canada, and Brazil (with approval expected in additional countries) for first-line treatment, in combination with fluoropyrimidine- and platinum-containing chemotherapy, of adults with locally advanced unresectable or metastatic HER2-negative G/GEJ adenocarcinoma and CLDN18.2-positive tumors [7–10]. In Japan, zolbetuximab is approved in combination with chemotherapy for patients with HER2-negative, CLDN18.2-positive, unresectable, advanced or recurrent gastric cancer [11]. Patients require CLDN18.2 testing to confirm eligibility for zolbetuximab treatment [7, 8].

The approved companion diagnostic (CDx) VENTANA CLDN18 (43-14A) RxDx Assay (Roche Diagnostics) [12] has been analytically and clinically validated in two phase 3 clinical studies of zolbetuximab, SPOTLIGHT (NCT03504397) [13] and GLOW (NCT03653507) [14]. In addition to the CDx, it is anticipated that CLDN18.2 testing will also be performed using independently established immunohistochemical (IHC) assays, known as laboratory-developed tests (LDTs), using the Ventana Benchmark IHC platform as well as other platforms, such as Leica and Dako, and other commercially available CLDN18 antibodies. Validation of individual protocols and information on inter-laboratory concordance of testing results is required to ensure reliable testing in clinical practice.

Laboratories performing diagnostic testing participate in quality assurance (QA) to assess the quality of their testing procedures [15]. External quality assurance (EQA) is a component of QA in which a laboratory’s testing procedures are compared with those of their peers and/or reference values [15]. Proficiency testing is a well-established EQA method for IHC biomarker testing. In proficiency testing, a laboratory performs analyses on pre-validated testing samples from an EQA provider and submits the results and the stained slides to the provider, who compares the results with pre-validated reference values and provides feedback to the laboratory on the accuracy of testing [15].

This prospective, in vitro, non-interventional study was designed to accompany an independent proficiency test (IPT; one of three phases of EQA) and descriptively analyze the results of CLDN18.2 IHC testing in gastric cancer tissue samples by laboratories in the DACH region (Germany, Austria, and Switzerland) using LDTs and/or in vitro diagnostic (IVD)-labeled assays independently established in their institutions. The aim was to describe the concordance of LDTs and IVD-labeled assays for CLDN18.2 IHC with the reference value of the samples. In addition, assay performance and scoring accuracy by pathologists (e.g., discordance and inter-LDT variability) were explored. Supporting reliable testing for this biomarker is essential to ensure appropriate identification and implementation of treatment targeting CLDN18.2 in patients with gastric cancer [15].

## Methods

The EQA proficiency test was conducted by Quality in Pathology (QuIP) GmbH, an accredited and experienced independent institution specializing in conducting EQA for IHC and molecular biomarker testing on tissue samples (including gastric cancer). QuIP was accountable for the scientific design, setup, conduct, and analysis of the proficiency testing, including provision of pre-defined samples to participating laboratories. The EQA proficiency test comprised the following phases: preparation, IPT/validation, and external laboratory proficiency testing (open proficiency test [OPT], including slide review).

Preparation entailed setup of EQA lead and panel institutes’ pathologists, selection of a sample panel by the EQA lead institute, and selection of control cases for IPT/validation. During IPT/validation, materials/cases were sent to the lead and panel institutes’ pathologists, results were received and discussed, and final cases for the OPT were selected (including only samples that were concordantly validated). Consensus scoring of samples, which was agreed upon prior to sample selection, was based on the cut-off used for enrollment in zolbetuximab phase 3 studies [13, 14], such that CLDN18.2 positivity was defined as ≥ 75% of tumor cells expressing membranous CLDN18 with ≥ 2+ (moderate to strong) staining intensity. The on-market CE-IVD VENTANA CLDN18 (43-14A) Assay (Roche Diagnostics) served as the gold standard.

The OPT involved the following steps: registration of interested pathology laboratories in the DACH region; provision of cases to participating laboratories; classification of cases (CLDN18.2-positive or CLDN18.2-negative) by participating laboratories using IVD assays or LDTs; submission of results and additional information to QuIP; and evaluation of results, including slide review (i.e., review by lead institutes and consultation with participants who encountered difficulties). Laboratories were provided with a lab manual containing information on materials, logistic process, and details of participation procedures (e.g., passing requirements, evaluation, data submission); furthermore, the EQA provider organized an online seminar explaining the guidelines for scoring and CLDN18.2 interpretation of stained slides. Ten samples with two slides per sample were supplied to each laboratory for staining with one or more IHC antibodies, followed by scoring and CLDN18.2 interpretation by a designated pathologist. The homogeneity of the samples and stability of the pre-validated reference values had been tested by the lead institute by staining reference slides with hematoxylin and eosin and for CLDN18.2 using the on-market CE-IVD VENTANA CLDN18 (43-14A) Assay (Roche Diagnostics). Laboratories submitted the results of their analyses to QuIP with percentage of stained tumor cells and a binary CLDN18.2-positive/negative result for each sample using the same cut-off used during IPT/validation and in the zolbetuximab phase 3 studies (i.e., ≥ 75% of tumor cells expressing membranous CLDN18 with ≥ 2+ [moderate to strong] staining intensity [13, 14]). Participants were asked to return the stained sections to QuIP. The lead institute evaluated them and, if necessary, provided guidance on optimization of staining.

For successful participation in the OPT, institutes had to correctly categorize (i.e., submit a result that was concordant with the reference value) for ≥ 90% of cases. Upon review by the lead institute, analyses were considered correct if they differed from the reference value due to tumor heterogeneity but no technical or interpretational errors could be detected, or if they were scored as “technically not evaluable” but there were justified deficiencies in the material. Overall proficiency of the OPT was considered acceptable if ≥ 80% of laboratories participated successfully.

## Results

Twenty cases with different CLDN18.2 expression levels were pre-selected for the IPT. Seven lead and panel institutes carried out the IPT using their individually established antibody protocols. The IPT showed a high concordance for 90% of the analyzed cases. Ten cases were selected for the OPT, and the reference value of every case was defined (Table 1). Results of the IPT for these 10 cases are presented in Supplementary Table 1.

**Table 1:** Overview of case selection and correctly analyzed cases

Case selection			
Case number: OPT	Case number: IPT	Reference values: CLDN18.2 status	Borderline case^a^
1	1	Negative	
2	2	Positive	
3	3	Negative	
4	4	Positive	
5	7	Positive	
6	13	Negative	
7	14	Positive	
8	17	Positive	X
9	19	Negative	
10	20	Positive	X
Correctly analyzed cases^b^			
Cases correct		Participants (%)	
10		36 (68)	
9		6 (11)	
8		3 (6)	
7		3 (6)	
6		2 (4)	
5		1 (2)	
4		1 (2)	
3		0	
2		0	
1		0	
0		1 (2)^c^	

^a^IPT cases 17 and 20 were defined as borderline cases with moderate to strong membranous CLDN18 staining in the range of 60%–90% of tumor cells

^b^Successful participation was defined as ≥ 9 cases correct. A total of 53 institutions participated

^c^One participant chose “technically not evaluable” for every case due to usage of an inappropriate antibody clone

CLDN18, claudin 18; CLDN18.2, claudin 18.2; IPT, independent proficiency test; OPT, open proficiency test

Fifty-four institutes registered for the OPT, and one institute resigned from the program during the testing process. There were 45 German institutes (including one resignation), five Austrian institutes, and four Swiss institutes, comprising 83%, 9%, and 7% of the institutes in the study. No data were available about the pathologists participating in the OPT, such as special interest in gastrointestinal pathology or biomarker analysis or general expertise in surgical pathology.

### Case-related results

Forty-two out of 53 (79.2%) institutes participated successfully (Table 1). One participant selected “technically not evaluable” for every case due to the usage of an inappropriate antibody clone (EPR19202 [Abcam]) and not observing sufficient staining to analyze the individual cases.

For case 8 (reference value: CLDN18.2-positive), two submissions were counted as correct even though they were assessed as negative due to tissue heterogeneity. The same applied for one submission for case 9 (reference value: CLDN18.2-negative), which showed positive staining in a few tumor cells. The main source of errors was false negative (FN) results, especially for cases 2, 5, 8, and 10 (Table 2).

**Table 2:** Overview of CLDN18.2 analysis in the OPT by case

Participant rating, n	Case 1	Case 2	Case 3	Case 4	Case 5	Case 6	Case 7	Case 8		Case 9	Case 10
	Negative	Positive	Negative	Positive	Positive	Negative	Positive	Positive (borderline)		Negative	Positive (borderline)
CLDN18.2-negative	**51**	12	**52**	2	6	**51**	3	8	*2*	**51**	6
CLDN18.2-positive	1	**40**	0	**50**	**46**	1	**49**	**42**		*1*	**45**
Technically not evaluable	1	1	1	1	1	1	1	1		1	2^a^

Values indicate the number of participating institutes selecting the category. Bold numbers indicate the validated reference value of the individual cases. Italicized values indicate submissions that were assessed as correct even if they differed from the initial reference value

^a^One participant had technical problems with case 10 because this case showed inhomogeneous, unspecific, and cytoplasmic staining patterns. In the review, this submission was considered evaluable and was therefore counted as incorrect

CLDN18.2, claudin 18.2; OPT, open proficiency test

### Key metrics

Cases 2, 8, and 10 had average percentage staining around the cut-off of 75% positive tumor cells with moderate to strong membranous staining (Table 3). Averaging scoring through all submissions of cases 2 and 8 would result in negative cases, as FN classification was assessed by the respective participants because of very weak staining intensity. Integrating the true positive (TP) evaluations exclusively and the median positivity confirmed cases 8 and 10 as positive cases closest to the cut-off value.

**Table 3:** Key metrics by case

Metric	Case 1	Case 2	Case 3	Case 4	Case 5	Case 6	Case 7	Case 8	Case 9	Case 10
	Negative	Positive	Negative	Positive	Positive	Negative	Positive	Positive (borderline)	Negative	Positive (borderline)
Reference value, %	17	89	26	98	93	26	93	84	49	81
Average % staining (total)^a^	27	74	38	91	86	24	85	72	39	76
Average % staining (TP or TN)^b^	27	85	38	94	92	20	93	84	35	81
Median % staining (total)^a^	30	83	40	95	95	10	90	80	40	80
Median % staining (TP or TN)^b^	30	85	40	95	95	10	95	80	40	80
Accuracy (%)	96	75	98	94	87	96	92	83	98	85
FPR (%)	2	—	0	—	—	2	—	—	0	—
FNR (%)	—	23	—	4	12	—	6	16	—	12
TPR (sensitivity) (%)	—	77	—	96	88	—	94	84	—	88
TNR (specificity) (%)	98	—	100	—	—	98	—	—	98	—

^a^Based on distinct proportions of moderate to strong CLDN18 membranous stained tumor cells. All values (i.e., also FPs or FNs) were included for the individual cases

^b^Based on distinct proportions of moderate to strong CLDN18 membranous stained tumor cells. Only TPs or TNs based on the reference values were included for the individual cases

CLDN18, claudin 18; FN, false negative; FNR, false negative rate; FP, false positive; FPR, false positive rate; TN, true negative; TNR, true negative rate; TP, true positive; TPR, true positive rate

Cases 2, 5, 8, and 10 showed the lowest accuracy and highest FN rate (FNR), as these cases were all validated as CLDN18.2-positive cases. Consequently, these cases also showed the lowest sensitivity/true positive rate (TPR), with case 2 being the most critical case.

Total key metrics confirmed the case-related observation that FN evaluations were the main factor for an incorrect assessment. In particular, the FNR (12%) in comparison to the false positive rate (FPR; 1%) underlines this observation. The true negative rate (TNR) was 99%, and the TPR was 88%. The negative predictive value was 84%, and the positive predictive value was 99%. The overall accuracy of 91% reflects the highest number of correct evaluations, with 10 correct categorizations.

### Antibody clones

**Fig. 1:**
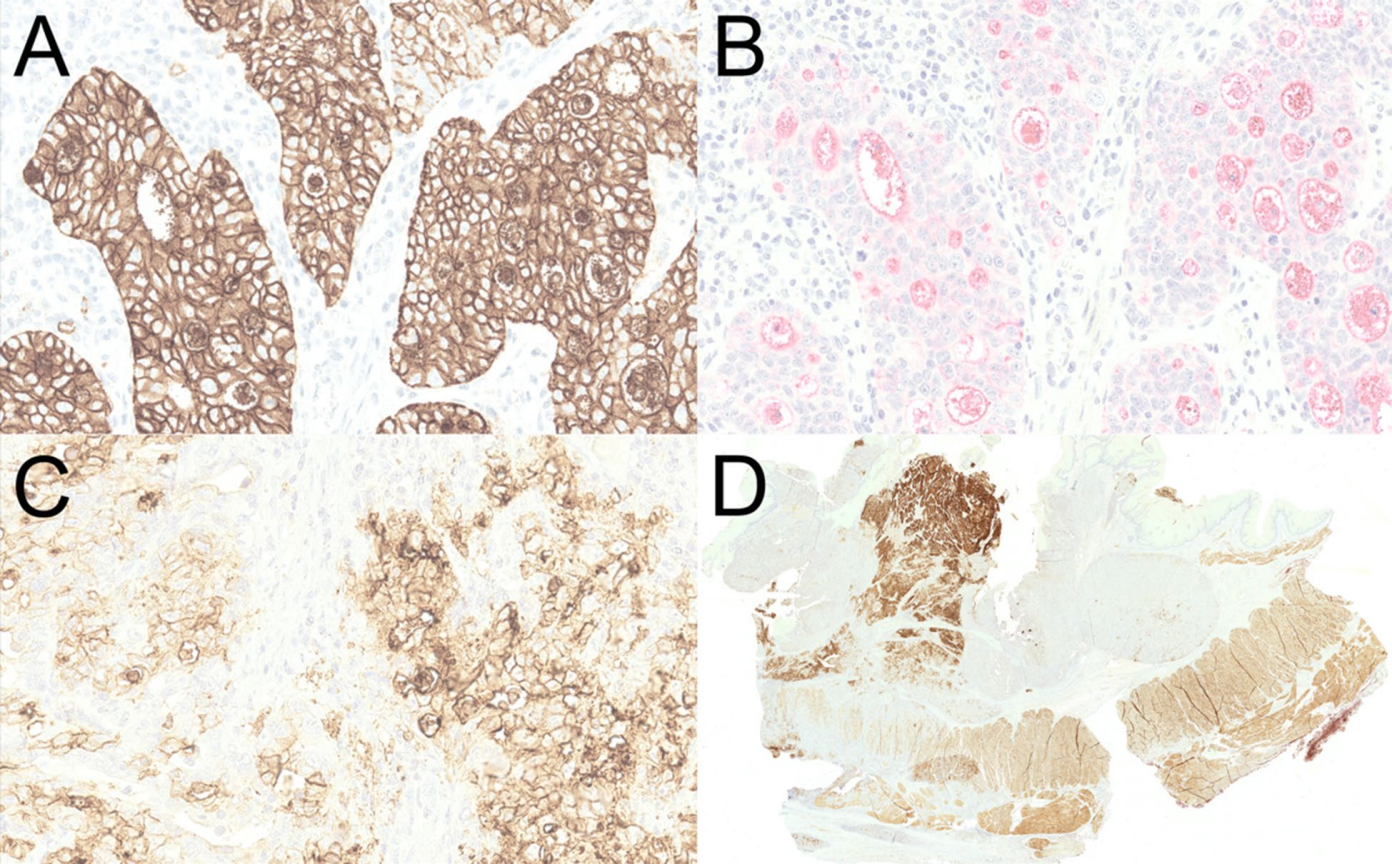
CLDN18.2 immunostaining with different antibodies. A strong (3+) and homogeneous membranous immunostaining of 100% of the tumor cells is shown with clone 43-14A from Ventana/Roche (**A**). The same case showed a weak (1+), barely detectable membranous staining with EPR19202 from Abcam in less than 50% of the tumor cells (**B**). A weak (1+) to moderate (2+) membranous staining was noted with ZR451 from Zeta/Zytomed in 100% of the tumor cells shown here (**C**). Note immunostaining of smooth muscle cells of the muscularis propria by ZR451 from Zeta/Zytomed (**D**). In addition, (**D**) illustrates heterogeneous staining for CLDN18.2 in gastric cancer with > 50% of the tumor area being negative. Original magnification 300-fold (**A**, **B**, **C**) and 60-fold (**D**). CLDN18.2, claudin 18.2

In total, 52 of the 53 participating institutes that submitted their final results provided information on the protocols used. Antibodies used for CLDN18.2 detection, success rates, and associated problems are summarized in Table 4. Representative immunostaining results with different antibodies are shown in Fig. 1. Thirteen of the 53 institutes (25%) had an isolated staining problem, whereas only three institutes (6%) had an interpretation problem for individual cases. Two institutes (4%) submitted results with a low-quality staining combined with an occasionally incorrect interpretation.

**Table 4:** Antibodies used for CLDN18.2 detection and associated problems detected in the review of individual participants

Antibody (manufacturer)	Total number of laboratories using antibody	Successful categorization (passing rate, %)	Problem analysis		
			Staining	Interpretation	Staining and interpretation
43-14A (Abcam)	2	2 (100)	—	—	—
VENTANA CLDN18 (43-14A) Assay (Roche Diagnostics)	28	27 (96)	1	2	—
ZR451 (Zeta/Zytomed)	9	5 (56)	7	—	1^a^
EPR19202 (Abcam)	5	0 (0)	5	—	—
PathPlus™ CLDN18 (LSBio)	3	2 (67)	—	1	—
HUAB10 anti-CLDN18.2 (not assignable)	1	0 (0)	—	—	1
Polyclonal rabbit (Invitrogen/Thermo Fisher)	2	2 (100)	—	—	—
MM02 (109915-MM02; Sino Biological)	1	1 (100)	—	—	—
RBT-CLDN18.2 (BioSB)^b^	1	1 (100)	—	—	—
Others/no information	1	1 (100)	—	—	—

^a^Participants who participated successfully with this clone submitted suboptimal stains

^b^RBT-CLDN18.2 could not be reviewed because the slides were not returned by the participant

CLDN18, claudin 18; CLDN18.2, claudin 18.2

The CLDN18 (43-14A) clone was used most frequently (VENTANA CLDN18 [43-14A] Assay [Roche Diagnostics], 28 laboratories; Abcam 43-14A clone, 2 laboratories). Overall, no significant technical or interpretation problems were observed with these antibodies. One LDT assay using the Ventana/Roche 43-14A antibody did not show sufficient staining and resulted in FN results for cases 2, 5, and 10; all additional submissions using the 43-14A clone showed optimal staining results. Two participants had problems interpreting a single case.

There were significant issues with staining quality associated with the ZR451 clone (Zeta/Zytomed; used by nine laboratories) and the EPR19202 clone (Abcam; used by five laboratories; Table 5). For the ZR451 clone (Zeta/Zytomed), lack of staining quality was associated with four unsuccessful submissions and suboptimal staining for participants with successful submissions, based on review of the stains by the lead panel. There was high-contrast membranous staining of the tumor cells, but also conspicuous strong cytoplasmic staining. Additionally, nonspecific weak staining of smooth muscle cells was observed. Thus, there is a risk that establishing this antibody and attempting to minimize the background staining will compromise the sensitivity of detection. Cases 2 and 8, in particular, showed high FN rates with the ZR451 clone (Zeta/Zytomed). The EPR19202 clone (Abcam) showed deficiencies across all five participants using it, resulting in multiple FN evaluations, particularly for cases 2, 5, 8, and 10. This antibody showed predominantly cytoplasmic and weak membranous staining and thus exhibited both sensitivity and specificity problems compared with other antibodies used in the OPT and in relation to the cut-off for CLDN18.2 positivity. Taken together, usage of the ZR451 clone (Zeta/Zytomed) and the EPR19202 clone (Abcam) contributed to 75% (9 out of 12) of the unsuccessful submissions; without them, > 80% of the participants were successful.

**Table 5:** Submissions for the EPR19202 (Abcam) and ZR451 (Zeta/Zytomed) antibody clones by case

Participant rating, n	Case 1	Case 2	Case 3	Case 4	Case 5	Case 6	Case 7	Case 8	Case 9	Case 10
	Negative	Positive	Negative	Positive	Positive	Negative	Positive	Positive (borderline)	Negative	Positive (borderline)
EPR19202 (Abcam)										
CLDN18.2-negative	**4**	4	**4**	1	3	**4**	1	3	**4**	2
CLDN18.2-positive	0	**0**	0	**3**	**1**	0	**3**	**1**	0	**1**
Technically not evaluable	1	1	1	1	1	1	1	1	1	2
ZR451 (Zeta/Zytomed)										
CLDN18.2-negative	**9**	6	**9**	0	3	**9**	1	5	**9**	2
CLDN18.2-positive	0	**3**	0	**9**	**6**	0	**8**	**4**	0	**7**
Technically not evaluable	0	0	0	0	0	0	0	0	0	0

Values indicate the number of participating institutes selecting the category. Bold numbers indicate the validated reference value of the individual cases

CLDN18.2, claudin 18.2

The PathPlus™ CLDN18 (LSBio) clone (used by three laboratories) showed an overall strong, high-contrast immune reaction. The distinction between strong cytoplasmic and membranous staining was occasionally problematic for this clone and led to discordant results for one participant. One participant reported that they used a HUAB10 anti-CLDN18.2 antibody, which could not be linked to a specific manufacturer. The specificity of this antibody was highly questionable, as it strongly stained smooth muscle cells, cells of the desmoplastic stroma, and inflammatory cells (nuclear and cytoplasmic). Individual tumor cells showed a strong cytoplasmic immune reaction and together led to an interpretation error. Two other antibodies, polyclonal rabbit (Invitrogen/Thermo Fisher; used by two laboratories) and MM02 (109915-MM02; Sino Biological; used by one laboratory), were assessed as optimal stains and did not lead to interpretational errors. The antibody RBT-CLDN18.2 (BioSB; used by one laboratory) could not be reviewed because the slides were not returned by the participant.

## Discussion

Biomarker testing is vital for identification and implementation of personalized cancer treatment with improved efficacy based on a patient’s genotype and biomarker expression [15, 16]. Accurate determination of CLDN18.2 expression will be important to confirm patient eligibility for zolbetuximab treatment for G/GEJ adenocarcinoma [7, 8] and for future CLDN18.2-targeted therapies. The CDx VENTANA CLDN18 (43-14A) RxDx Assay (Roche Diagnostics) is approved as the analytically and clinically validated assay for zolbetuximab [12, 17]. However, some institutions use LDTs and IVD-labeled assays, which are developed and validated in-house and are not approved by any regulatory bodies. This study examined the performance of LDTs and IVD-labeled assays for evaluation of CLDN18.2 expression in gastric cancer samples through proficiency testing, an established method to assess accuracy and identify issues contributing to inaccurate results [15].

Results of the OPT showed good overall concordance of CLDN18.2 evaluation in gastric carcinoma in the participating institutes. The 79.2% success rate of the OPT (42 of 53 institutes participating successfully) indicated a quality-assured diagnostic landscape. The narrow miss of the 80% threshold could be explained by the recent establishment of CLDN18.2 testing in laboratories, and the main reason for discordance was staining problems associated with antibody selection (Table 4). Participant interpretation errors were detected but were not a significant problem.

The main factor for an accurate analysis is the choice of an appropriate antibody clone. The antibody that is purchased and validated as an LDT is at the discretion of the institution. Procurement costs, the staining system available at the institution (Dako, Leica, or Ventana), and available literature sources are influencing factors. At the time of the OPT, there was only one published international comparative study, which compared three different antibodies and staining systems [18]. With the exception of the Ventana/Roche 43-14A and PathPlus™ antibodies, no other antibody used in the OPT had previously been examined in a similar comparative study.

The 43-14A clone was associated with optimal staining results. It detects both CLDN18.2 and CLDN18.1 isoforms, but this is not considered problematic for staining of primary gastric tumors because CLDN18.1 is predominantly expressed in the lung, whereas CLDN18.2 is specific to normal and malignant gastric mucosa [16, 19]. The EPR19202 clone demonstrated poor analytical performance and could not accurately detect CLDN18.2. Overall, the staining was too weak, leading to sensitivity and specificity problems and unsuccessful participation of all institutes using this antibody. The ZR451 clone also demonstrated poor performance, such that four out of nine institutes using this antibody did not participate successfully. This antibody showed high cytoplasmic background staining, and attempts to optimize the background staining could lead to loss of sensitivity and incorrect classification. Taken together, use of these two clones contributed to 75% (9 out of 12) of unsuccessful submissions. Without the EPR19202 and ZR451 clones, > 80% of participants were successful, indicating that LDTs can deliver quality equal to the on-market CE-IVD VENTANA CLDN18 (43-14A) Assay (Roche Diagnostics) and lead to accurate determination of CLDN18.2 status.

This study had limitations. The number and geographic location of participating institutes do not provide a complete overview of the expected diagnostic landscape for CLDN18.2 testing in gastric cancers; hence, the results cannot be generalized completely. It is anticipated that additional institutes in the DACH region will establish CLDN18.2 testing, as zolbetuximab is now approved for use in Europe [8]. Ongoing QA will be needed to monitor the testing landscape for institutes that adopted CLDN18.2 evaluation later and continually monitor laboratories that already routinely test. Given that the evaluation of individual protocols is limited by the relatively small number of users, the results do not provide definitive conclusions but give an impression of potential problems and pitfalls. Lead and panel institutes were preselected by QuIP and not included solely based on objective criteria. Guidelines for selection included scientific expertise, accreditation or certification, and experience with other newly established proficiency testing schemes.

In conclusion, proficiency testing was carried out successfully. Overall, CLDN18.2 testing was successfully established in pathology laboratories of the DACH region, with a 79.2% success rate that increased to > 80% after excluding two antibodies associated with a high risk of FN stains. Staining quality attributed to appropriate antibody selection is the major factor necessary for successful analysis. Ongoing QA may assist in the establishment of appropriate protocols ensuring accuracy in CLDN18.2 testing.

## Supplementary Information

Below is the link to the electronic supplementary material.

Supplementary file1 (PDF 44 KB)

## Data Availability

Anonymized data are available upon reasonable request from the corresponding author.

## Abbreviations

CDx: Companion diagnostic
CLDN18: Claudin 18
CLDN18.2: Claudin 18.2
DACH: Germany/Austria/Switzerland region
EQA: External quality assurance
FN: False negative
FNR: False negative rate
FP: False positive
FPR: False positive rate
G/GEJ: Gastric or gastroesophageal junction
HER2: Human epidermal growth factor receptor 2
IHC: Immunohistochemistry/immunohistochemical
IPT: Independent proficiency test
IVD: In vitro diagnostic
LDT: Laboratory-developed test
OPT: Open proficiency test
QA: Quality assurance
QuIP: Quality in Pathology
TN: True negative
TNR: True negative rate
TP: True positive
TPR: True positive rate
