# Supplementary material for: Concordance of laboratory assays for claudin 18.2 in gastric cancer tissue samples: independent proficiency testing and a descriptive non-interventional study
Source: Virchows Arch. 2025 Jun 28;487(5):1117–25. doi: 10.1007/s00428-025-04138-x (PMC12647276; doi:10.1007/s00428-025-04138-x)
Supplement: Supplementary file 1 — Supplementary file1 (PDF 44 KB) [file 428_2025_4138_MOESM1_ESM.pdf]

SUPPLEMENTARY INFORMATION

Virchows Archiv

Concordance of laboratory assays for claudin 18.2 in gastric cancer tissue samples: independent proficiency testing and a descriptive non-interventional study

Christoph Röcken,[1] Anne Kathrin Höhn[2], Jens Neumann[3], Hans-Ulrich Schildhaus[4], Stephan Singer[5], Till S. Clauditz[6], Alexander Quaas[7], Korinna Jöhrens[8]

[1]Department of Pathology, University Hospital Schleswig-Holstein, Campus Kiel, Arnold-Heller-Str 3, 24105 Kiel, Germany; [2]Universität Leipzig, Haus G, Liebigstraße 26, 04103 Leipzig, Germany; [3]Institute of Pathology, Medical Faculty, Ludwig Maximilian University Munich, Thalkirchnerstr. 36, 80337 Munich, Germany; [4]Discovery Life Sciences & Institute of Pathology Nordhessen, Germaniastraße 7, 34119 Kassel, Germany; [5]Institute of Pathology, University Hospital Tübingen, Liebermeisterstr. 8, 72076 Tübingen, Germany; [6]Center for Diagnostics, Institute of Pathology, University Medical Center Hamburg-Eppendorf, Martinistraße 52, 20246 Hamburg, Germany; [7]Institute of Pathology, Cologne University Hospital, Kerpener Str. 62, 50937 Köln, Germany; [8]Qualitätssicherungs-Initiative Pathologie QuIP GmbH, Reinhardtstraße 1, 10117 Berlin, Germany

Corresponding author

Christoph Röcken

Email: christoph.roecken@uksh.de

Supplementary Table 1 Results of the IPT for the 10 cases selected for the OPT

Case	Result	Reference value	LP (43-14A LDT)	P1 (43-14A IVD)	P2 (43-14A IVD)	P3 (43-14A IVD)	P4 (43-14A IVD)	P5 (43-14A LDT)	P6 (43-14A IVD)
1	CLDN18.2 status	Negative	Negative	Negative	Negative	Negative	Negative	Negative	Negative
	% staining	17%	50%	5%	5%	10%	15%	n.d.	15%
2	CLDN18.2 status	Positive	Positive	Positive	Positive	Positive	Positive	Positive	Positive
	% staining	89%	100%	90%	80%	85%	90%	n.d.	90%
3	CLDN18.2 status	Negative	Negative	Negative	Negative	Negative	Negative	Negative	Negative
	% staining	26%	50%	20%	20%	30%	25%	20%	20%
4	CLDN18.2 status	Positive	Positive	Positive	Positive	Positive	Positive	Positive	Positive
	% staining	98%	100%	100%	95%	100%	90%	n.d.	100%
5	CLDN18.2 status	Positive	Positive	Positive	Positive	Positive	Positive	Positive	Positive
	% staining	93%	100%	90%	95%	90%	90%	n.d.	90%
6	CLDN18.2 status	Negative	Negative	Negative	Negative	Negative	Negative	Negative	Negative
	% staining	26%	50%	40%	40%	10%	15%	10%	20%
7	CLDN18.2 status	Positive	Positive	Positive	Positive	Positive	Positive	Positive	Positive
	% staining	93%	100%	95%	90%	95%	90%	n.d.	90%
8	CLDN18.2 status	Positive	Positive	Positive	Positive	Positive	Positive	Positive	Positive
	% staining	84%	100%	80%	90%	75%	80%	n.d.	80%
9	CLDN18.2 status	Negative	Negative	Negative	Negative	Negative	Negative	Negative	Negative
	% staining	49%	50%	60%	70%	40%	50%	40%	30%
10	CLDN18.2 status	Positive	Positive	Positive	Positive	Positive	Positive	Positive	Positive
	% staining	81%	80%	80%	90%	80%	n.d	n.d	75%

CLDN18.2, claudin 18.2; IPT, independent proficiency test; IVD, in vitro diagnostic; LDT, laboratory-developed test; LP, lead panel; n.d., not determined; OPT, open proficiency test; P, panel
